# Cancer nutrition and rehabilitation—its time has come!

**DOI:** 10.3747/co.v15i3.244

**Published:** 2008-06

**Authors:** M.R. Chasen, A.P. Dippenaar

**Affiliations:** * Clinical Director, Cancer Nutrition and Rehabilitation Program, McGill University, Montreal, QC; † Medical student, Pretoria University, South Africa

**Keywords:** Rehabilitation

## Abstract

Cancer is a systemic disease that can affect nearly every organ in the body, resulting in a progressive loss of organ function. That loss of function may be initially slow, having minimal effect, or it may be rapid, resulting in more dramatic changes.

The usual medical management of patients with cancer has focused more specifically on the administration of cytotoxic treatments. These treatments can potentially eradicate or minimize the tumour, but they may also have toxic side effects that in turn can also affect the patient.

Cancer rehabilitation is a process that assists the individual with a cancer diagnosis to obtain optimal physical, social, psychological, and vocational functioning within the limits created by the disease and its treatment. The McGill Cancer Nutrition and Rehabilitation (cnr) program developed as a result of the ever-increasing demand for a focus on addressing individual cancer patients and their needs, as well as on achieving optimal tumour-related outcomes. Using an interdisciplinary approach, the cnr’s global objective is to empower individuals who are experiencing loss of function, fatigue, malnutrition, psychological distress, and other symptoms as a result of cancer or its treatment to improve their own quality of life. All team members—experts in their respective fields—assess all patients. At a subsequent team discussion and planning meeting, a specific 8-week program is designed for each patient. The hoped-for outcome for the cnr program is primarily to empower patients to “take control” or to enable them to improve their own quality of life. This article reviews the philosophy of the cnr’s approach and the roles played by the various members of the team.

## 1. INTRODUCTION

Cancer may develop in almost any area or system within the body 1. The neoplasm not only directly affects the region in which it arises; it can metastasize to distant sites, causing a variety of complications at those sites. Disruption of metabolism and endocrinology may directly or indirectly influence any number of bodily systems 1,2. The many complications that the cancer causes can affect a patient’s quality of life in many domains—physical, social, psychological, and work-related.

The medical management of patients with cancer has usually focused on the administration of cytotoxic treatments to potentially eradicate or minimize the tumour burden. These treatments may be extremely toxic to the body. Various treatment modalities are often a cause of altered functioning, affecting daily occupational routines 1. Co-administered medications, used to control symptoms or to treat adverse events associated with treatment modalities, can produce their own adverse events.

The primary aim of treatment has been to cure patients of their disease. However, the American Society of Clinical Oncology has emphasized to its members the importance of recognizing the point in the illness trajectory when treatments should focus more on symptom management and psychosocial support for the patient whose tumour is not curable. That approach takes into account the cultural, religious, and social belief systems that can also influence the patient’s perspective on his or her disease and on the illness experience 1,3,4.

## 2. CANCER REHABILITATION

Cancer rehabilitation is a process that assists the individual with a cancer diagnosis in obtaining optimal physical, social, psychological, and vocational functioning within the limits created by the disease and its treatment. An imperative in accomplishing these goals is a coordinated multidisciplinary team approach that addresses the potential rehabilitation needs of the individual from the time of the cancer diagnosis onward.

Historically, the concept of cancer rehabilitation stems from an integral component of *The* [U.S.] *National Cancer Act of 1971.* That legislation declared cancer rehabilitation to be an objective, and it directed funds toward the development of training programs and research projects. In 1972, the U.S. National Cancer Institute sponsored the National Cancer Rehabilitation Planning Conference. That conference identified the following four objectives for the rehabilitation of cancer patients:

- Psychosocial support
- Optimization of physical functioning
- Vocational counselling
- Optimization of social functioning

The McGill Cancer Nutrition and Rehabilitation (cnr) program developed as a result of the ever-increasing demand for a focus on addressing individual cancer patients and their needs, as well as on achieving optimal tumour-related outcomes. The McGill cnr program was the brainchild of Dr. Neil MacDonald, who, after many years of caring for patients with advanced cancer, initiated a clinic for patients who were experiencing anorexia and cachexia. This clinical syndrome, consisting of extreme weight loss and physical dysfunction, is the result of abnormal cytokine production associated with cancer.

The cnr assumes that the patient and the patient’s environment are the center toward which all interventions are directed. The cnr recognizes that each patient is an individual and therefore that each patient requires different types and levels of intervention. Accurate history-taking and meticulous symptom assessment are key components in directing appropriate interventions that treat the most distressing symptoms. Interventions may include medical treatments (chemotherapy and all other prescriptions given to counteract disease or treatment-induced side effects—constipation, for example); nutritional (dietary advice, nutraceutical prescription) and physical (exercise, ergonomic assessment) support; and possibly psychosocial evaluation (distress thermometer) and treatments (cognitive behavioral therapy). Many, if not most, patients also use therapies such as natural health products, acupuncture, massage, and *qigong,* to name a few. These therapies also need to be evaluated in the context of the cnr.

Depending on the needs of the individual patient and family, members of the rehabilitation team may include any or all of physicians, oncology nurses, dietitians, physical and occupational therapists, social workers, psychologists, recreational therapists, vocational therapists, case managers, patient coordinators, chaplains, and relevant volunteers. The effects of the illness extend beyond the patient into the social network and larger community. These extended community connections, also including the primary caregiver and other family members, may at times be considered part of the cnr team. They also may require specialized therapy to manage the consequences of the illness.

Research in clinical oncology has focused mainly on determining the best approaches to evaluate anticancer treatment—that is, drugs, radiation, and surgical procedures—so as to determine the traditional endpoints of disease-free and overall survival. Over recent decades, studies have increasingly evaluated the effects of other interventions such as psychosocial, exercise, and nutritional interventions not only on the traditional endpoints, but also on health-related quality of life 5 and a variety of other patient-reported outcomes. Several reviews and meta-analyses have demonstrated subjective patient benefits (for example, quality of life, symptom control, coping mechanisms) for such approaches 1,6,7.

The global objective of the McGill cnr program is to use an interdisciplinary approach to empower individuals who are experiencing loss of function, fatigue, malnutrition, psychological distress, and other symptoms as a result of cancer or its treatment to improve their own quality of life.

### 2.1 Patient Assessments

The principles of rehabilitation dictate that, before any intervention is implemented, a full and appropriate assessment of the patient’s needs, available support systems, and potential obstructions to optimization of personal functionality must be performed:

- A baseline assessment highlights current physical, nutritional, emotional, domestic, and vocational problems that the patient may be experiencing.
- Regular ongoing assessments, including a global screening, identify needs in the following areas:
  - Functionality (activities of daily living, physical functioning, and mechanical functioning such as swallowing; functioning of prostheses and stomas is also vital to the patient’s functionality)
  - Fatigue
  - Nutrition
  - Symptoms (for example, pain, sleep, loss of appetite and weight, dyspnea)
  - Psychosocial wellbeing (sexuality, depression, anxiety)
  - Vocational wellbeing (plans to return to gainful, meaningful employment)
  - Spiritual wellbeing
- Caregiver assessment determines the status and needs of the patient’s immediate caregiver:
  - The caregiver’s role and the effects of being a caregiver
  - Communication between friends and family
  - Ability to prepare nutritious, appealing food
  - Potential for developing complicated grief
  - Ability to be an advocate
  - Ability to assist in care and management of the patient

### 2.2 The Interdisciplinary Team

Because of the variety of problems that patients may potentially face during the course of their illness, rehabilitation requires an interdisciplinary team approach 1,2,6. Individual expertise is combined to achieve the maximum effect on the patient’s life and to target the specific problems identified by the patient. The patient’s needs determine the team members that have to be involved, and treatment plans are individualized to meet each patient’s unique and specific situation 3–5.

The interdisciplinary approach of the McGill cnr assists a cancer patient in obtaining maximum physical, social, and psychological functioning within the confines that the disease, or the treatment, or both, have created. The cnr strives to assist individuals to return to the highest level of functioning and independence of which they are capable and to achieve their goals.

#### 2.2.1 Physician

Clinicians on the interdisciplinary team include physicians from several specialties, such as primary care physicians, surgeons, radiation oncologists, and medical oncologists. The clinical examination of the patient and the evaluation of the patient’s physical wellbeing is handled by a physician. In addition, evaluation of events and assessment of the efficacy of medication is performed at regular intervals. The patient’s participation in, and access to, trials of new agents is an important component of physician responsibility. Another important role is facilitation of communication between referring physicians and the rehabilitation team so that a relationship of trust can be built. A good trust relationship allows for better and more effective overall treatment for the patient, and fewer conflicting opinions between referring physicians and the rehabilitation team.

#### 2.2.2 Nurse

The pivot nurse is the first point of contact between the cnr team and the patient, and thus has the task of primary patient triage and screening a. Many patients may not be suitable for the program because of general health that is too poor, geographic inaccessibility, or a lack of enthusiasm to participate in the program. The nurse must evaluate each case individually after a thorough examination of the medical chart and an in-depth interview with the patient.

The pivot nurse then presents the relevant history to the other members of the cnr team in advance of a full team evaluation. The nurse also performs a full assessment of the main symptoms or other issues presented by the patient and his or her family.

Nurses play the main role in communicating with patients outside of the clinic; they facilitate the team–patient liaison through regular follow-up telephone calls and visits. The patient’s navigation of the hospital system (for example, organizing radiologic or hematologic examinations, and locating the necessary departments) is made easier by the nurse. Similarly, the nurse makes referrals to community services for home visits and help.

Psycho-educational groups, which provide patients and their families with the knowledge necessary to deal with the challenges that face them, are organized weekly and facilitated by the nurse. Over the 8 weeks of the program, the nurse teaches and coaches patients in effective strategies to self-manage symptoms more effectively, to navigate and manage within today’s complex health care system, and to broker and advocate for care and needed help.

The nurse typically functions as an extension of other members of the team, frequently assisting with treatment interventions provided by other professionals. Because nurses typically have extensive contact with patients and families, they may be most aware of the family’s emotional stress and adjustment issues 3,4. Nurses sometimes function as counsellors, providing substantial emotional support to patients and their families.

#### 2.2.3 Dietitian

Appropriate diet and adequate nutrition are important factors in a patient’s rehabilitation. Weight loss in a patient with cancer carries with it the consequences of poorer prognosis, increased toxicity from chemotherapy, increased fatigue, and breakdown in social communication, especially at mealtimes. These effects can substantially influence the patient’s overall quality of life. The dietitian’s role is to evaluate the patient’s current nutrition status and to provide recommendations regarding specific dietary needs. Many patients need to identify the foods and smells that cause adverse symptoms such as nausea. Adequate education regarding the prevention and treatment of constipation is also a vital function, as is appropriate mouth care. Dietary supplements and alternative foods are discussed and prescribed. Dietitians also teach family members about the importance of appropriate diet in successful rehabilitation 8,9.

#### 2.2.4 Physical and Occupational Therapists

Improvement of the patient’s physical condition, functional capacity, and ability to carry out daily activities are the main goals. Through exercise programs and other physical goal-directed activities, the building of strength, improvement of endurance, and reduction of fatigue will result in improved mobility for the patient^3^,^4^. The physical and occupational therapists on the team provide assistance with activities of daily living 6 such as dressing, bathing, cooking, working, and other basics that may be difficult to continue because of the effects of the disease or the chemotherapy 6, 10. In so doing, the patient gains greater patient autonomy and self-efficacy and reduces dependency on caregivers or welfare.

The physical therapist initially evaluates the patient’s muscle strength, mobility, and joint range of motion 3,4. The treatment interventions that the physical therapist provides include therapeutic exercises to maintain or increase range of motion, endurance, and mobility training (for example, transfers, gait, stair climbing) 10.

The occupational therapist initially evaluates a patient’s ability to carry out activities of daily living such as washing, dressing, preparing meals, working, driving, or performing leisure activities^3^,^4^,^6^. A Simmonds Functional Scale Assessment, which allows for a better understanding of the day-to-day functional ability of the patient, is also performed 11. Education on energy conservation, including the use of compensatory techniques, how to plan and set priorities, and the use of adaptive equipment are part of the therapeutic armamentarium. Patients are also supported and assisted in developing new vocations or returning to previous employment, albeit with assistance that may be temporary or permanent.

#### 2.2.5 Psychologist

Patients and their families often have a number of psychological and adjustment issues related to the illness and its treatment The psychologist assesses and treats social, emotional, and mental functioning through patient and family education and counselling for stress, anxiety, and depression management 3,4. Using cognitive behavioral therapy, existential psychotherapy, couples therapy, and support groups, the psychologist helps the patient to adjust to actual, perceived, and potential losses.

Anxiety and depression may also be managed with techniques such as mindfulness-based meditation. The goal of consulting the psychologist is to maximize the benefit that the patient derives from rehabilitation^3^,^4^.

The psychologist not only acts as a member of the rehabilitation team, but also assists other members of the team with psychological issues stemming from the management of patients or their family members. This important contribution should occur on a regular, planned basis.

#### 2.2.6 Social Worker

The role of the social worker varies substantially depending on the individual’s needs. Often, the social worker provides counselling to patients and families regarding emotional support, community resources, finances, lifestyle changes, and their participation in treatment 3. Sometimes, the social worker may lead support groups and actively assist in discharge-planning activities, such as arranging for home-care services, for transfer to other health care settings, and for introduction to community-based care systems 4.

#### 2.2.7 Clinic Coordinator

The vital contribution of the clinic coordinator is the synchronization of appointments for patients and the handling of all the clerical and administrative work needed for the smooth daily running of the clinic.

### 2.3 Patient Trajectory Through the Clinic

#### 2.3.1 Which Patients?

Patients that are referred are those who, as compared with their level before diagnosis, are experiencing changes in

- appetite (with or without associated weight loss);
- physical functioning, such as walking;
- fatigue; and
- coping with the consequences of their disease.

All new-patient visits begin in the morning. On arrival, patients are weighed and handed several questionnaires for self completion:

- The Edmonton Symptom Assessment Scale 12
- The Patient-Generated Subjective Global Assessment 13
- The Brief Fatigue Inventory 14
- The Distress Thermometer 15
- The Dyspepsia Severity Symptom Index 16

The various professionals see each patient during this first visit. Each member of the team evaluates the patient individually for 30 minutes. During that time, the patient remains in a single location, and team members move between patients. Thereafter, the patients return home with the assurance that the nurse, following an in-depth discussion between team members that takes place in the afternoon, will contact them.

The post-examination discussion is a pivotal meeting during which the various team members present their findings. These are challenged or supported by other members, thereby allowing every patient and his or her unique problems and difficulties to be reviewed. The patient is thus considered as a whole person. Planning of an individualized program then follows, and the program is instituted. An initial summary of findings and the resulting plan is communicated to the referring physician.

Once accepted into the program, patients have biweekly exercise sessions with the physiotherapist. A fortnightly (or more frequent, if needed) visit to the dietitian, occupational therapist, nurse, physician, and other relevant team members is scheduled. If judged necessary or if specifically requested by the patient, a detailed psychological assessment is undertaken, and specific therapy is given. Weekly psycho-educational sessions are held for patients, their families, and staff.

At the end of the 8 weeks, a full repeat of the baseline assessment is conducted. For patients that require still more formal supervision in any component of the cnr program, a personal referral to other rehabilitation units such as the Comprehensive Health Improvement Program is made. All patients are also referred back to their original physician with a full follow-up summary and recommendation.

#### 2.3.2 Outcomes

The hoped-for outcome for the cnr program is primarily to empower patients to “take control” or to enable them to improve their own quality of life. Empowerment can be accomplished by educating the patients regarding

- prescribed medications and their purpose;
- improved or maintained nutritional intake and weight;
- increased engagement in exercise, walking, and daily activities;
- decreased symptoms of anxiety and depression;
- increased self-efficacy of caregivers in care processes; and
- increased self-efficacy for communication within the family and within social and health care networks.

All of the foregoing assistance results in improvements in quality of life:

- Better understanding of how and when to take medications to prevent or terminate symptoms
- Improved efficacy and pleasure in eating, and a return to mealtime as a positive social interaction
- A return to work and confidence in independently performing daily activities
- A more thorough understanding of the causes and handling of mentally distressing emotions
- Efficacy in accessing all available support systems—financial, social, educational, and recreational

Anticipated long-term outcomes include

- justification of medication;
- a healthy lifestyle, including regular exercise, correct eating, and healthy thoughts;
- improved familial and other interpersonal relationships;
- a decrease in visits to health care providers and the emergency room; and
- a decrease in days of hospital stay.

## 3. CONCLUSIONS

The cnr program offers a multidimensional, holistic treatment approach emphasizing the patient as an individual. Treatment interventions therefore provide attention to detail for each individual patient. Regular team meetings and discussion regarding the patient’s progress over the 8-week program ensure that effective communication between team members is maintained. Constant education of patients in regard to their own disease and treatment is a central theme that allows the patients to feel empowered and not helpless and hopeless. A cnr should be incorporated into most, if not all, cancer treatment programs.

## Footnotes

a Eades M, Skrutkowski M, Carney S. Key to continuity of care: the pivot nurse within the cancer nutrition rehabilitation (cnr) interdisciplinary team. Presented at the 19th Annual Canadian Association of Oncology Nurses Conference; October 28–31, 2007; Vancouver, BC.
